# ‘We are looked down upon and rejected socially’: a qualitative study on the experiences of trafficking survivors in Nepal

**DOI:** 10.3402/gha.v8.29267

**Published:** 2015-11-18

**Authors:** Pranab Dahal, Sunil Kumar Joshi, Katarina Swahnberg

**Affiliations:** 1 Department of Health and Caring Sciences, Linnaeus University, Kalmar, Sweden; 2 Department of Community Medicine, Kathmandu Medical College, Kathmandu, Nepal

**Keywords:** sexual trafficking, Nepal, reintegration, victimization, stigma

## Abstract

**Background:**

The successful reintegration of sexual trafficking survivors into Nepalese society is challenging. This paper aims to explore the trafficking process, abuses faced during sexual slavery,and the challenges faced by women and girl survivors for successful reintegration.

**Method:**

This exploratory study used qualitative methods to identify that poverty, illiteracy, lack of opportunities, and varied social stigma initiate the victimization process, and continuity of this vicious circle increases the risk for (re)entrapment.

**Result:**

The reasons for sexual trafficking have also become the reasons for restricting survivors from opportunities for growth and mainstreaming.

**Conclusion:**

Non-existent support systems, detachment from familial ties, being outcast by society, and an uncertain livelihood make reintegration difficult for survivors.

The recorded trafficking history of Nepalese women and girls for sexual exploitation dates back to the fall of the Rana regime in Nepal (1950s), when ethnic girls from the hills serving as servants and concubines were sold to the brothels in India (1). Although there have been many debates on the number of trafficked persons, almost all studies show a strong presence of the sexual trafficking phenomenon in Nepal. The official estimate suggests that the recorded number of trafficking cases is on the rise, with 5,500 known cases in the year 2010 increasing to 11,500 cases in 2011 (2). A report by the Human Rights Watch in 1995 had already produced an estimate of 200,000 women and girls involved in forced prostitution in India. The conflicting numbers suggest that the actual magnitude of women and girls who are victims of sex trafficking in Nepal is still unknown and this assumption remains valid to date (3, 4). The estimates are thought to be speculative and are often based on observations and anecdotal information rather than on scientific evidence, which is partly a reflection of the clandestine and illegal nature of sex trafficking (5).

The underreporting of sexual trafficking and the absence of uniform data management systems have produced different numbers, and for a country like Nepal finding an exact estimate is difficult. Moreover, on a global scale, the numbers have been conflicting. The global data suggest that 58% of human trafficking includes sexual trafficking of women and girls, equivalent to 4.5 million persons who are trafficked for commercial sexual exploitation. Yet another estimate suggests that 2.5 million children, women, and men are lured or forced across international borders every year and many more are trafficked within their home countries, put to work against their will, often under deplorable and unsafe conditions, and held captive by physical, psychological, or financial threats (6–8). Not only are the estimates and numbers on trafficking a contested phenomenon, but a general consensus on the definition of trafficking has also been difficult to reach. There is no consistent use of the term *human trafficking* and no consensus on what the term comprises or how it relates to issues of forced labor, slavery, and exploitation (8). This paper uses the definition of trafficking adopted by the SAARC Convention on Preventing and Combating Trafficking in Women and Children for Prostitution (2), as it focuses more on girls and women involved in forced sex labor, which is also the subject of this study. The convention defines trafficking as ‘the transportation, selling or buying of women and children for (forced) prostitution within and outside a country for monetary or other considerations with or without the consent of the person subjected to trafficking’.

Human trafficking in Nepal is often cited as the result of poverty and destitution. The migration decisions of an individual leading to trafficking are often influenced by poverty, disintegration of family, experiences of violence and abuse, false promises of a better life or of marriage, promise of a tourist holiday, and so on (9). The subordinate position of women has aggravated gender-based inequalities with increased risks of vulnerabilities, abuse, and trafficking. Human trafficking in the region has been referred to as an integral component of the traditional economy and the cycle of movement of people in South Asia (10). The trafficking process in Nepal, as identified by Hennink and Simkhada (5), is characterized by four methods of trafficking: through brokers, independent migration to urban areas, deception or false marriage, and force/abduction. Until now, trafficking in Nepal has been perceived exclusively as the sexual exploitation and slavery of women and girls in Indian brothels. A shift has emerged in the patterns of human trafficking in Nepal with the opening of the borders to foreign labor migration and the rapid internal displacements caused by the cross-cutting issues interlinked with poverty, unemployment, gender discrimination, social exclusion, and globalization (11).

The number of annually returning survivors is still unknown in Nepal. Furthermore, since most of these returning survivors do not use rehabilitation and shelter homes, determining the actual numbers of returning trafficking survivors is difficult. Reintegration is described as the process of reunification with economic viability and social acceptance (12). However, reintegration does not seem to be an easy process; trafficking survivors are considered shameful and are further stigmatized by their families and communities, making reintegration difficult (12, 13). Successful reintegration is a complex process of approval, consisting of social, psychological, and economical components. The social components of rehabilitation are intended to mainstream trafficked survivors who are considered to be marginalized or stigmatized women. The psychological components are meant to enhance the self-esteem of trafficked survivors, and the economic components focus on the economic empowerment of the survivors of trafficking in finding an alternate livelihood (14).

Reintegration remains challenging in Nepal due to continued social stigma and discrimination against survivors (15, 16). Many trafficking survivors returning from sex industries suffer from severe post-traumatic effects caused by inhumane practices faced at the brothels (17), and the complete denial and rejection on all fronts of life in their own society increase the risks of re-victimization. Banovic and Bjelajac (18) reported that during sexual slavery survivors are often physically abused and raped, have their movements restricted, are denied food and water, are tortured or drugged for absolute obedience, and face penalties for breaching any established rules. Chen and Marcovici (12) found that fear of being stigmatized is a hindrance to successful reintegration of trafficked survivors in Nepal. The survivors often become a topic of disagreement, and the victimization process starts with seclusion and lack of any opportunities for the survivors. A study by Joshi et al. (19) on female trafficking survivors from Nepal confirmed that reintegration becomes challenging with dysfunctional families; consequently, most survivors return to the sex trade as a means of survival, in order to hide from family and friends. In her study on trafficked survivors in the United States, Shigekane (20) explains that even if survivors of trafficking are settled in their community, they face challenges like a sense of terror, helplessness, and lack of confidence in appearing in public, which result in psychological trauma. The threats from society and pressures from one's family and relatives add extra emotional and psychological strains, increasing threats of abuse and re-victimization. Stigmatization by the social environment, discouraging reintegration, is often cited as the primary cause of re-trafficking. Trafficked survivors are frequently rejected and shunned by their families or communities for having been forced to work as a prostitute, sexually abused, failing to return with the promised income, or for leaving a debt unpaid (21).

## Aims of the paper

This paper explores the trafficking process, abuses faced during sexual slavery, and the challenges faced by women and girl survivors in successful reintegration after returning to Nepal. The paper addresses issues relating to rehabilitation and reintegration, highlighting the complexities of social, cultural, gender, and economic aspects, whereby women are victimized both during the trafficking and the reintegration stages. Trafficking occurs as a result of violence and exploitation of women; this paper also hinges on identifying factors nurturing the trafficking problems in Nepal. Finally, the paper explores the reintegration process and the challenges faced by survivors in successful mainstreaming.

## Methodology

This study was exploratory in nature, using qualitative descriptive methodology, including 10 in-depth interviews and one focused group discussion (FGD) with trafficking survivors. A purposive sampling method was used for this study. A local nongovernmental organization (NGO) working with trafficking survivors helped to establish contact with the informants. The inclusion criteria for this study were survivors who were transnationally trafficked, had a history of living in safe homes, and were above 18 years of age. The perceptions, beliefs, and experiences of trafficking survivors were collected using preformulated interview guides with open-ended questions by the first and second authors of this paper, both of Nepali origin and male. The interview and FGD guides were developed by the study team to incorporate themes of inquiry on the familial contexts of survivors, trafficking process, sexual slavery at the brothel or private home, rescue process, and reintegration. The in-depth interviews were semi-structured, and a more relaxed, informal interviewing method was adopted for rapport and trust building. The interviews with the trafficking survivors lasted for 60 to 90 min. The FGD was conducted among survivors to gain new insights but also to triangulate information collected during the interviews in order to increase validity. Both interviews and the FGD were conducted privately at the safe home of the NGO. A voice recorder was used to record information during the interviews and FGD. The in-depth interviews and FGD were conducted in the local language (Nepali) and later translated into English. A translator for other Nepali languages was available but none of the respondents felt the need, as they were comfortable in expressing themselves in Nepali. The English translation process focused more on getting relevant meaning than exact translations of the verbal information received. The information collected from the field notes and recordings were transcribed and later divided into different themes to impart relevant meanings. The themes represented a patterned response within the data set (22). The themes from the data were organized into meaningful groups to get information on the phenomenon (23, 24). The major identified themes were categorized into themes of trafficking and reintegration; challenges and subthemes in relation to gender, stigma, poverty, coping, problems, and health were assessed and are presented in the ‘Results’ section of this paper.

### Ethical considerations

The research followed the WHO ethical and safety recommendations for research on domestic violence (25) and the Declaration of Helsinki (DoH), focusing on privacy and confidentiality, informed consent, and guided research protocol for research involving human subjects. The safety and confidentiality of the informants were established by conducting private interviews, whereas the FGD aimed toward the more social issues of sexual trafficking and reintegration. Participants were adequately informed about the aims and methods of the research, and following DoH ethics every single participant had equal rights to refuse to participate in the study or to withdraw their consent to participate at any time without reprisal. Written approvals were also exchanged between the researcher and the respondents to ensure the anonymity and confidentiality of the information and the respondents’ identities. Prior verbal consent was obtained from all participants for voice recording of the interviews and the FGD. Ethical approval was obtained from the ethical review committee of Kathmandu University, Kathmandu Medical College, for conducting this study.

## Findings

### Informants’ backgrounds

The ages of the trafficking survivors ranged from 19 to 50 years. The youngest trafficking age was identified as 14 years, with most of the survivors being trafficked during their early teens and mid-20s. The survivors originated from the rural villages of the Sindhupalchwok, Kavrepalanchowk, Bhaktapur, Dhankuta, and Bardiya districts of Nepal. Out of ten respondents, three were illiterate, while the others had some level of education but not beyond 7 years of schooling. Kathmandu, the capital, was identified as the departure city for most of the survivors for cross-border trafficking. The duration of forced prostitution for the survivors ranged from 2 weeks to 7 years. Rescue by the police was identified as the most common method, who set survivors free from the brothels. Six survivors reported going to their families and friends immediately after their return, while four survivors spent 1 to 3 months in safe homes during a transition period.

### The trafficking process

Deceit, false hopes, and abduction were found to be the most common methods used for trafficking. All of the survivors communicated that unmet needs and dreams of overcoming poverty were the major reasons for falling victim to false promises. The gender disparity originating from popular socio-cultural beliefs also led to differential treatments and practices for the survivors earlier in their life, reinforcing the subordinate positions of the women and increasing their vulnerabilities and easy accessibility to the pimps and agents for abuse and trafficking.

I was working in Kathmandu as a housemaid, miles away from my home situated in Charikot, Sindhupalanchowk district. I met a woman, a frequent visitor and a family friend of the house. As our closeness grew, she offered me an idea to start a business by bringing garments from India to sell in Nepal. She lured me, saying that there was more money to make from the business than serving in a house as a maid. I was keen to earn more money and settle well; I wanted to get rid of poverty and her idea of a business hit me hard.

Hopes for foreign employment with large sums of money have also been used as a method to trap women. It was known that a decent paying job was promised by the employment agencies for which the survivors also reported to have paid commissions worth thousands of Nepalese rupees for the service, only to later find out that they had been tricked and sold for prostitution. The idea of foreign employment is found to mask transnational sexual trafficking, which exists in Nepal.

I was born in the far western region of Nepal. Raising three children in poverty was not easy. To support my family and children, I decided to go abroad for a job. I managed to find an opportunity through an employment agency; I was told I would be working as a cleaner in a hospital or a school or at the airport in Kuwait. I had signed a paper to pay the sum of forty thousand Nepalese rupees for the job, to be later deducted from my salary.

The networks of these sexual traffickers are powerful and have also infiltrated the state apparatus, including the security services and immigration department. It has become almost impossible to pass through immigration without adequate documentation and work permits, but traffickers have managed to establish a system where these obligatory procedures have been breached.

I was surprised to find a policeman escorting me at the airport in Nepal while boarding a flight to Lebanon. He took me directly to the aircraft, and I did not have to go through any of the immigration checks.

The victimization process starts by creating false hopes and dreams, and in most cases the dream of escaping poverty is used as powerful bait by the brokers. The survivors, on the other hand, with dire needs are readily convinced. The brokers specifically target mostly vulnerable women who are far away from any established social support systems.

I was born in Kavrepalanchowk, and my parents died when I was still a child. My brothers started neglecting me once they got married and at the age of twelve, I fled to Kathmandu … as I could not receive care and support at my brother's home. Upon coming to Kathmandu, I realized life was not easy. Finding food and shelter became a big problem for me. I met a stranger who was willing to help me find a job. I never knew where I would be working, but I was told about a place called Raxaul (India). I had already started earning a living in Kathmandu with petty jobs as a helper, cleaner, and domestic worker at different places. I was persuaded for 3–4 months about this job in India. Finally, I agreed, as I was assured that this job would allow me to earn more. I was taken to a hotel in Kathmandu where I was offered food, and I remember nothing before waking up in Raxaul.

The social positioning of women being treated like a commodity also reinforces their vulnerability, leading to their continued exploitation. The denial of the right to choices and having no access to decision making, as well as unequal positioning and severe gender discrimination, have positioned women and girls on the bottom rung of the social ladder in Nepal. It becomes easy for opportunist brokers to further exploit these exploited masses.

I was fourteen when my sister's wedding procession arrived at our home. To our surprise, my sister ran away with her lover on the day of her marriage. The wedding did happen, but the bride was changed. I had to replace my sister as a new bride but against my will and consent. After the marriage, I was always taunted and got continuous scolding from my in-laws, as I was not their real bride.

Gender-based discrimination, providing unequal positioning for females, also increases the risk of being abused. Depriving females of education and dowry-related mistreatment have also been identified as the beginning of the victimization process. Women and girls are thus found to grasp at any opportunities in front of them masked with false promises, increasing their vulnerability to trafficking.

I was married when I was twelve. Belonging to a poor family, I didn't come to my husband's home with a dowry or gifts, and it became the root cause of a bitter relationship with my in-laws. The physical assault and beatings started right after three days of my marriage. I continued to stay in that home and gave birth to three children.

The involvement of close family members in trafficking was also identified. Declaring a husband–wife relationship at border checkpoints provided an easy escape from interrogation by officials. It was ascertained that brokers even married women or cultured a close romantic relationship with them in order to win their confidence and to smuggle them to the brothels.

I was working in a brick kiln when I got into a close relationship with a worker from India. Although he was much older than me, I never had any problems. He invited me to visit his home in India and I happily followed him. While crossing the border, we were interrogated about our visit and relationship, and he said that we were married and going to visit relatives in India although we were not married.

Abduction was also identified as a method of trafficking. In this form of trafficking, the survivor is unaware of the destination and coercion is used. The survivors are mostly drugged to prevent retaliation and suspicion from others.

I was forcefully abducted from my village when I was fourteen. I woke up in the train not knowing where I was going. As I regained consciousness, I was given juice to drink and it made me more lethargic and sleepy. I finally opened my eyes to discover that I had already been sold and the place I was living was called a *kothi* (brothel).

### Period of sexual slavery

The lives of the survivors during sexual slavery at the brothels speak of severe abuse. Arriving at a new place, without any acquaintances, and being subjected to continuous sexual slavery broke down the women. It was noted that the repeated physical violence and unwilling monotony of being just an object of sexual gratification caused the development of sudden suicidal tendencies in most of the trafficked survivors.

I landed in Hong Kong in the evening and was immediately taken to a hotel. After reaching the hotel, my passport was seized and then I was told that I had been sold for prostitution. At times, I used to hide in the warehouse filled with shoes and cover myself with the boxes. I felt that committing suicide was better than selling my body.

The survivors are cut off from the outside world and forced to live in conditions similar to a prison. The captors, on the other hand, use all kinds of methods to continue the enslavement of the trafficked; any documents of identification and money remaining to the survivors were immediately seized, and their movements were always monitored and restricted.

For three months, I didn't know that I had already been sold. There were almost seventy women from different countries in the building where I was kept. For three months, I kept nursing four to five children and learning the language. At the end of the three months, I was shifted to another room but in the same building. Since then, I had to offer sexual services to six to seven men during a day. After exactly a week, I thought of killing myself rather than living a life in hell. The living room of the house was ornamented with spears and swords; I managed to grab a spear and was determined to kill myself by plunging a spear in the electric circuit box. I remember waking up at the hospital with bandages below my waist. I stayed in the hospital for a month receiving treatments for my injuries and burns. After being discharged from the hospital, I was taken to the same house and kept under even stronger captivity on the twenty-fifth floor. I decided to escape using a rope made out of curtains, bedsheets, wire and quilt covers. I managed to reach the ground but my hands, knees, and legs were hurt badly and I was bleeding profusely. I walked into the Nepalese embassy, bleeding heavily with just a cloth wrapped around my body.

The survivors were subjected to severe inhumane practices. The consequence of continued physical abuse and repeated abortions also increase health risks. The survivors have to remain at the mercy of their captors if they develop any known symptoms of diseases, and spending money for health checkups does not fall in the priority list for these captors.

After reaching Lebanon, my passport was immediately seized and I started living as a prisoner in a big house. Later on, I realized that I was in a house where I had to sexually serve the owner and guests visiting the house. I found out that I had been sold for $1000. I felt like killing myself when I had to sleep with the drunken Arab men, but the constant thought of my child back home stopped me from committing suicide. I got pregnant twice and both times I was taken to a hospital for abortions. Later on, I was sent back to Nepal after two years with nothing but a developing child in my womb.

The sale and resale of the survivors was also reported to be a common practice at the brothels in India. Uncooperative survivors and additional income from the trade are known to be the prime reasons for reselling the trafficked individuals. For the survivors, the resell meant nothing more than a change in location and new clients, with the continuity of the same routine job of sexual enslavement.

I was imprisoned for some time, as I forcefully reacted against sleeping with any male client. I used to get regular beatings from the brothel owner in a small dark room. To mask my cries and squeals, songs were played at the highest volume using a cassette player. Despite all my protests, I had to submit my body to the clients and sleep with them. This continued for two to three months until the brothel owner was tired of my uncooperative behavior. I was resold to another brothel for a sum of fifty thousand Indian rupees. After being sold again to a new place, I was forced to sleep with twenty to twenty-five clients per day.

### Coming home

For most of the trafficking survivors, rescue from the sex industry occurred through police raids, help from clients, or captors releasing the survivors due to age, health conditions, or debt clearance. Few survivors were able to free themselves from the brothel through personal escape plans with help from others (fellow survivors, clients, brothel guards) to run away. Rehabilitation centers for the trafficking survivors were operated by NGOs, providing accommodation and food, health checkups, and training in a few livelihood skills.

Upon returning to Nepal, I was taken to a rehabilitation center for sexual trafficking returnees; seven or eight young trafficked women joined me at the safe house. The women were in a state of terrible shock and crying most of the time. The next day, I was taken to the hospital for checkups, I was diagnosed with several physical and mental problems, and I ended up staying in the hospital for almost six months treating my diseases. Even today, I am taking prescription medicines. It has been almost one and a half years, and I am out of the rehabilitation center and I am still not able to work due to my fragile health condition.

The question of the sustainability of rehabilitation efforts is pivotal. The returnees remain thankful to NGOs for the immediate support and care; however, at the time of release from the shelter home, they did not find themselves to be equipped with all the necessary skills and preparedness for reintegration into the society on their own. NGOs, on the other hand, with resource constraints, mainly target short-term support services, limited to three months. The returnees were found to be unaware of the service providers for future help, lacked adequate occupational skills to start a new life, and felt too weak to voice their concerns.

I find the rehabilitation of trafficked women supported by NGOs to be a bit incomplete, as it never ensures what will happen to the survivor once she is out of the center. Also, the programs are largely externally funded and when the money stops, there is no way to provide any support to the survivors. The rehabilitation program has to focus on equipping the returnees with skills so that they can stand on their own feet.

The returnees find it close to impossible to find proper ways to reintegrate into their own society. Continued social stigma, lack of support, and limited opportunities for finding any means of survival cripple and isolate them to a maximum.

Even a glass of drinking water from the hands of a returnee is considered to be impure and unholy; their presence at any cultural or religious events is considered to be a bad omen. The trafficking returnees are always looked down upon and rejected socially. The short stay at the rehabilitation centers does provide some security, but it becomes completely different after coming out into the real world.

The trafficking survivors feel that the social understanding of trafficking returnees is that they are degraded and corrupt individuals, who should be outcasts and tormented to extremes. A constant reminder of the survivor's past increases further abuse; the returnees have to remain as silent spectators in unfriendly surroundings.

I felt completely alone when my community knew that I was a trafficking returnee and also HIV infected; they even barred me from watching television in a common room. I was always a topic of discussion, and people even feared speaking to me.

The social denial and rejection often leaves returnees to face hardship in continuing their life. Without proper skills and adequate knowledge, they are forced to use manual labor as their only means for survival. It was reported that survivors choose to work at places far from their origin; they often tend to choose new places where their identities are not known to people. This strategy is devised to get away from the stereotyping and stigmas. It was reported that four of the trafficked survivors returned to prostitution and the sex trade, as they could not find any other means for survival. The difficulties faced by the trafficking survivors often compel them to self-inflict the re-victimization processes.

I had to return to prostitution to get money for the abortion of the fetus I had carried from Lebanon. None of my family or relatives know what had happened to me, but I have heard them gossiping about me behind my back.

### The way forward

This section discusses the overall experiences of survivors during the reintegration process and their viewpoints on how things actually should be, for ending the status quo and mainstreaming their needs and agenda. It was reported that reintegration efforts are not complete until the trafficking returnees find a means of survival to live a dignified life. The barriers to successful reintegration, according to the trafficking survivors’ experiences, include continuous stereotyping, leading to isolation and limited access to opportunities.

The trafficking returnees have to be equipped with skills so they can earn a living other than entering the sex profession again. The re-victimization process can only stop when the women have abilities and opportunities to lead their life.

Raising awareness and sensitizing the public to end all discrimination against trafficking survivors will surely take time, but any effort to mainstream the concerns and needs of the survivors is a good start. The social stigmas and belief systems embedded within communities cannot be changed overnight, and with the absence of any support structures it becomes difficult for the survivors to voice their concerns.

For successful reintegration, the basic survival needs of trafficked survivors have to be ensured. Their short-term needs and long-term survival mechanisms have to be provided for a safer transition and to sustain the reintegration process. There is an immediate need to find solutions to safeguard the interests and needs of survivors, in reducing stigmas and the associated risks to give them a chance to be more productive in ending all the re-victimization risks. The surviving women also need to empower themselves to put an end to uncertainty, passivity, and risk reduction. Sensitization and awareness on the social front are also imperative for safeguarding the survivors’ interests.

Providing skills through trainings would help women to gain financial security and help them to stand on their own feet. If I had received any formal training or any vocational skills, I would have been earning more money to meet my needs rather than relying on the hardship of manual labor.

The vulnerability of survivors infected with HIV and the added responsibilities of taking care of their children makes coping difficult. The lack of information about available services and the absence of adequate support mechanisms to address the physical and traumatic conditions of survivors further add to the difficulties for the returnees. The continued stigma toward the survivors and their children isolates them completely, secluding them from opportunities and reinforcing the victimization cycle. The vicious cycle of abuse and (re)victimization also affects the children, as they find themselves barred from opportunities for education and face discriminatory treatment from society.

People in remote villages still believe that HIV spreads through speaking, touching, and even sitting close to the infected person. Lack of knowledge, being stigmatized, and lack of acceptance have increased hatred for survivors with HIV.

The lack of opportunity and access to education, health, and legal services are some of the problems faced by the survivors’ children. All of the survivors showed greater concern for the safety, security, and growth of their children; however, the conditions do not seem to be favorable, as the children of survivors continuously face lesser opportunity for growth than other children.

## Discussion

The successful reintegration of trafficking survivors is challenging. Mahendra et al. (13) reported that the acceptance of trafficked returnees is difficult, and the stigma associated with prostitution leaves them in isolation. On the other hand, trafficked survivors also fail to construct their own identities as other than a sexual object, which further escalates their isolation and rejection (26). This non-acceptance at the community level and the inability to construct new identities makes reintegration challenging, as preparedness at the community level and among survivors are both absent. The stigmatization of trafficking survivors goes beyond the survivors themselves; the survivors’ families also have to be prepared to face stigmas associated with trafficking. In their research study, Hennink and Simkhada (5) reported that family members also start rejecting the surviving returnee in order to maintain their social status. The denial and rejection caused by the stigma force survivors to flee from their communities, often right back to the sex trade (27).

After going through the traumatic experience of sexual slavery, the survivors cannot remember the person they used to be, nor can they perceive a life away from this occupation, which often creates a degraded self-image (28). Successful reintegration requires opportunities for the returnees, with access to rights and services (29). Brukitt (30) explains that trafficking survivors need to find answers to questions such as ‘where can I go,’ ‘who am I,’ ‘what can I do,’ and ‘how do I identify myself in society’. Finding meaningful answers to these questions will enable returnees to reintegrate with ease, but finding answers to these questions is not easy and not only the women's responsibility until the social preparedness of acceptance on all fronts is guaranteed. When societies and families reject the individuals, reintegration can be extremely challenging (31). Denial and social rejection were reported to be the start of the re-victimization process by the survivors in this study.

Rehabilitation and reintegration programs require multifaceted approaches, involving a variety of actors. Reintegration efforts must simultaneously address the physical, psychological, traumatic, behavioral, social, and economic issues encountered by the trafficking survivors. Furthermore, the synergies of state actors, NGOs, local agencies, communities, and families have to be put together to produce visible results (17). It is noteworthy that rehabilitation and reintegration are different phenomena, and NGOs have been specifically targeting the rehabilitation aspect with immediate support rather than holistic reintegration of the surviving returnees. The cycle of re-victimization can only end when sustainable reintegration of the survivors is ensured, with education and employment prerequisites. These needs can be satisfied only through comprehensive and institutionalized programs including detection and identification, rehabilitation, reintegration, and sustainable social inclusion (18). The circle of violence and exploitation may be terminated only when a survivor is identified and subsequently re-socialized and reintegrated through an institutionalized support system (18). Comprehensive approaches to reintegration should take a survivor-oriented approach, satisfying the standards of process orientation (emotional healing and overcoming trauma) and effect orientation (emotional stabilization and social inclusion), as well as change in policies and enhancing survivors’ protection (32).

Gender inequality is a primary cause for sexual trafficking in Nepal, and this inequality also leads to several challenges for the effective reintegration of sexual trafficking survivors. Women and girls remain a vulnerable population, and the risk for victimization increases with poverty, lack of education, low socioeconomic status, limited employment opportunities, marginalization, corruption, weak governance, and discrimination (33). The vulnerability and marginalization of women and girls puts them at the mercy of the powerful patriarchal belief systems and practices offering them subordinate positions. The dominant Hindu value system in Nepal also reinforces the traditional patriarchal views regarding women's sexuality and makes provisions for institutionalized prostitution (the Badi system) prevalent in Nepal (34).

Exploring the firsthand experiences of trafficking survivors was challenging. The interviews took a long time, as it takes time to gain trust; in addition, the survivors needed to travel back in time to share their personal accounts and experiences of sexual slavery and servitude. Identifying and reaching the survivors was also not easy, as survivors tend to mask their identities. The methodological challenge of identifying and reaching trafficked survivors was facilitated by the involvement of NGOs working with the trafficked survivors. The respondents did not have difficulty in sharing information with the male researchers, as they felt that the information provided by them would contribute to the greater good of the population at risk. The personal experiences of the survivors varied immensely; however, the emerging commonalities of the trafficking process and reintegration issues ensured the trustworthiness of the information received. The major shortcoming of this study lies in its limited scope of generality of the findings; however, this study explores an overview of practices and trends in transnational sex trafficking as well as the reintegration challenges faced by survivors in Nepal.

## Limitations

The paper only focuses on the experiences of trafficked women and girls and does not reflect the views and opinions of wider communities. The participation and inclusion of opinions of community, family, and opinion leaders were beyond the scope of this paper.

## Conclusions

The study confirms that trafficking in Nepal is a complex cross-cutting issue linked to poverty, unemployment, gender discrimination, exclusion, and migration. Unlike conventional thought linking sex trafficking only to India, this study has identified trafficking survivors returning also from Kuwait, Lebanon, and Hong Kong. The study finds that women face severe abuse and violence during their stay at brothels. Their return to Nepal, following their rescue, also fails to realize the hopes of the survivors, as they remain alienated; furthermore, for returnees infected with HIV the situation is even more difficult. A reintegration process that fails to give an identity to the trafficking survivor is incomplete; the survivors in this study confirm that measures for successful reintegration should also take into consideration the creation of one’s identity, other than as a sex trafficking survivor. The lack of established support systems and diminished access to different livelihood opportunities, as well as a lack of skills, have made it difficult for survivors to create new identities for a new life. Short-term rehabilitative and reintegration efforts provide short-term relief, but the lack of sustainable practices fails to provide adequate security and opportunities for survivors. Furthermore, the lack of stringent policies safeguarding returnees’ needs has increased the risk of abuse and started the victimization process all over again, often forcing survivors to return to the sex trade and live a secluded life, away from their families and friends.
